# The pleiotropic effects of the glutamate dehydrogenase (GDH) pathway in *Saccharomyces cerevisiae*

**DOI:** 10.1186/s12934-018-1018-4

**Published:** 2018-11-01

**Authors:** P. Mara, G. S. Fragiadakis, F. Gkountromichos, D. Alexandraki

**Affiliations:** 1 Department of Chemistry, University of Crete, Voutes University Campus, 71003 Heraklion, Crete Greece; 2 Department of Biology, University of Crete, Voutes University Campus, 71003 Heraklion, Crete Greece; 3 Institute of Molecular Biology & Biotechnology, FORTH, Nikolaou Plastira 100 GR-70013, Heraklion, Crete Greece; 4 Present Address: Woods Hole Oceanographic Institution, Woods Hole, MA 02543 USA; 5 Faculty of Biology, Biocenter, Ludwig-Maximilians-University of Munich, Großhaderner Str. 2, 82152 Planegg-Martinsried, Germany

**Keywords:** Glutamate dehydrogenase, *GDH1*, *GDH2*, *GDH3*, Ammonium assimilation, GABA shunt, ROS-mediated apoptosis, Chromatin regulation, Nitrogen catabolite repression, *S. cerevisiae*

## Abstract

Ammonium assimilation is linked to fundamental cellular processes that include the synthesis of non-essential amino acids like glutamate and glutamine. In *Saccharomyces cerevisiae* glutamate can be synthesized from α-ketoglutarate and ammonium through the action of NADP-dependent glutamate dehydrogenases Gdh1 and Gdh3. Gdh1 and Gdh3 are evolutionarily adapted isoforms and cover the anabolic role of the GDH-pathway. Here, we review the role and function of the GDH pathway in glutamate metabolism and we discuss the additional contributions of the pathway in chromatin regulation, nitrogen catabolite repression, ROS-mediated apoptosis, iron deficiency and sphingolipid-dependent actin cytoskeleton modulation in *S.cerevisiae*. The pleiotropic effects of GDH pathway in yeast biology highlight the importance of glutamate homeostasis in vital cellular processes and reveal new features for conserved enzymes that were primarily characterized for their metabolic capacity. These newly described features constitute insights that can be utilized for challenges regarding genetic engineering of glutamate homeostasis and maintenance of redox balances, biosynthesis of important metabolites and production of organic substrates. We also conclude that the discussed  pleiotropic features intersect with basic metabolism and set a new background for further glutamate-dependent applied research of biotechnological interest.

## Background

Ammonium assimilation into carbon chains follows specific biosynthetic routes that lead to the production of non-essential amino acids including glutamate. Initial observations in bacteria showed two major mechanisms that can be used for the production of glutamate. The glutamine synthetase (GS) and glutamate synthase (GOGAT) (GS–GOGAT) mechanism that occurs when cells grow in low ammonia concentrations and the glutamate dehydrogenase (GDH) pathway that has a lower energy cost and is used by the cells in excess of ammonium and phosphate [1].

**Fig. 1:**
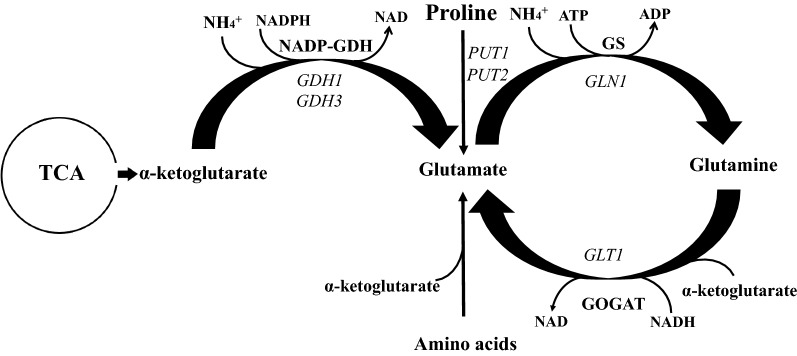
Glutamate production and nitrogen assimilation in *S. cerevisiae*. The figure was adapted from Fig. 1 [7]. *GDH1* glutamate dehydrogenase 1, *GDH3* glutamate dehydrogenase 3, *PUT1* proline oxidase, *PUT2* Δ1-pyrroline-5-carboxylate dehydrogenase, *GLN1* glutamine synthetase (GS), *GLT1* NAD(+)-dependent glutamate synthase (GOGAT), *TCA* tricarboxylic acid cycle

Central nitrogen metabolism in *Saccharomyces cerevisiae* hosts the same two conserved mechanisms for glutamate production [2–5]. GS–GOGAT pathway in yeast has a marginal contribution in glutamate synthesis both in fermentation and respiratory conditions, while the GDH pathway has the prominent role [5, 6]. Yeast strains lacking the GDH route, present a proline utilization pathway (PUT) that can contribute together with the GS–GOGAT in glutamate biosynthesis and nitrogen assimilation [7]. The relative contribution of PUT is being dictated by the nitrogen sources [7]. To our knowledge, *S. cerevisiae* is the only organism having three pathways for glutamate synthesis (Fig. 1) with the produced glutamate responsible for the 85% of the total cellular nitrogen and glutamine for the remaining 15% [8–10].

In this review, we summarize the current state of knowledge on the GDH pathway in *S. cerevisiae* and we aim to present the pleiotropic effects of the pathway in addition to its well-described role in glutamate metabolism. We discuss the findings presented in recent research and primary literature papers and we make a coherent argument about the topic.

## The GDH pathway in *S. cerevisiae*

In yeast, glutamate can be synthesized through the activation of the GDH pathway when ammonium is present in abundant levels. This can be achieved through the activation of MEP permeases (Mep1, Mep2, Mep3) that facilitate the entrance of ammonium in the cell and its retention in the cytoplasm [9, 11]. Under sufficient ammonium levels, the GDH pathway catalyzes the synthesis of glutamate using α-ketoglutarate and ammonium through the NADP linked action of GDH (1):

1 $$\alpha{\text{-ketoglutarate }} + {\text{ NH4}}^{ + } + {\text{ NAD}}\left( {\text{P}} \right){\text{H}}^{{}} \to {\text{ Glutamate }} + {\text{ NAD}}\left( {\text{P}} \right)^{ + }$$

The NADP-dependent GDH enzyme in yeast is encoded by *GDH1* and *GDH3* [12]. *GDH1* and *GDH3* are paralogous genes with *GDH3* originating from an ancestral event of whole-genome duplication [6, 13] or interspecies hybridization [14]. Despite the high conservation of *GDH1* and *GDH3*, Gdh1p exhibits higher utilization rates of α-ketoglutarate under glucose conditions compared to Gdh3p [15, 16]. This observation makes Gdh1 the primary (hyperbolic) NADP-GDH enzyme and Gdh3 the cooperative NADP-GDH isoform in the GDH pathway of *S. cerevisiae.* A recent study addressed the question whether the different utilization rates of α-ketoglutarate by Gdh1p and Gdh3p correlate with their evolutionary origin [6]. The authors compared the NADP-GDH activity of *S. cerevisiae* with that in closely related yeast species. The kinetic properties of NADP-GDH activity that derived from yeast species with either constitutively respiratory metabolism, or intermediate fermentative capacity were similar to the Gdh1 and Gdh3 isoforms of *S. cerevisiae* and complemented the total NADP-GDH activity. Based on this it was concluded that the different utilization rates of α-ketoglutarate by Gdh1p and Gdh3p were independent of their evolutionary origin [6].

In terms of localization, Gdh1p is found in the cytosol and the nucleus as opposed to Gdh3p that is localized in the mitochondria and the nucleus [17, 18]. This different localization seems to be evolutionarily retained due to the urge of a wise cellular exploitation of α-ketoglutarate that in many organisms acts as a signal and coordinates carbon and nitrogen metabolism [19]. Growing evidence shows that modulation of the intracellular α-ketoglutarate levels could constitute an important mechanism of metabolic control that can also interfere with many physiological processes [6, 20]. Enzyme purification experiments showed that Gdh1p and Gdh3p are hexamers (a6 50 kDa oligomeric structure) with the in vivo total NADP-GDH pool being a quite dynamic mixture of Gdh1p and Gdh3p monomers [15]. It was observed that under glucose fermentative growth the pool consisted mainly of Gdh1p monomers [12, 15]. *GDH3* is a glucose-repressed gene and consequently the presence of Gdh3 protein in the pool was very low [15, 21–23].

The allosteric regulation of NADP-GDH activity is influenced by α-ketoglutarate and NADP, and not by small molecules (e.g. GTP, AMP) or amino acids as has been reported for other GDH proteins, including human GDH [24].

### *GDH1* transcriptional regulation and phase-specific degradation of Gdh1 protein

The regulation of *GDH1* under glucose conditions is performed by nitrogen catabolite repressor (NCR)-sensitive activators, Leu3p and activators exclusive for respiratory growth such as the HAP complex that coordinates nuclear and mitochondrial gene expression [21, 25, 26]. Under ethanol conditions, *GDH1* derepression is mediated by the Gcn4 and Hap4 transcriptional activators and is amplified by Gln3 [21, 27]. Experiments measuring the β-galactosidase activity of *GDH1* promoted-*lacZ,* and nucleosome scanning assays in cells grown in glucose or ethanol with ammonia as nitrogen source, found that *GDH1* transcription occurs throughout all growth phases in yeast [6, 15, 21, 23]. This is achieved through different members of the SAGA remodeling complex that modify the chromatin for *GDH1* expression under different carbon sources [21]. The constant expression of *GDH1* implied that its transcription proceeds normally during the different growth phases including the diauxic shift, when yeast cells reprogram their metabolism to enter the respiration phase. However, during the post-diauxic shift, the Gdh1p/Gdh3p ratio decreases and most of the NADP-GDH activity is attributed to Gdh3p [15]. The decrease of the NADP-GDH activity in ethanol growing cells was initially referred to be controlled through post-translational modifications [28] that could modulate the proportion of Gdh1p versus Gdh3p monomers that constitute the NADP-GDH pool [21]. Indeed, proteomic studies revealed that Gdh1p can be a potential target of ubiquitin attachment [29, 30]. Additional studies showed that Gdh1 protein is subjected to a “stationary phase-specific degradation” that occurred at the diauxic shift [23]. Through a series of point mutations and protein quantification experiments, it was concluded that Lys-426 (K426) in the C-terminal box is essential for the observed stationary phase-specific degradation of Gdh1p [23].

This phase specific degradation of Gdh1p and its substitution by Gdh3p in the NADP-GDH activity pool seems to be favorable under glucose deprivation. As discussed above, Gdh1p utilizes α-ketoglutarate at higher rates compared to Gdh3p, and contributes to glutamate production when yeast cells ferment, and thus are in the exponential phase. Glucose deprivation signifies the entrance of the yeast cells into a stationary-phase survival mode. The transition from fermentation to stationary phase conditions is accompanied by a dramatic growth reduction and a sharp drop in protein synthesis characteristic for stationary phase survival [31, 32]. In addition, transitioning to stationary phase, requires yeast cells to recruit different defense mechanisms that will protect them from ROS-mediated damage that influences lifespan [33]. Lee et al. [23] suggested that the consequence of significant decrease in protein synthesis will be also reflected as significant decrease in amino acid synthesis, since a variety of amino acids, including glutamate, become unnecessary in stationary phase cells. Therefore, it may be more beneficial for yeast to substitute Gdh1p with Gdh3p through the phase-specific expression of *GDH3* and the simultaneous degradation of Gdh1p [23]. The authors observed that Gdh3p seems to be more suitable for the stationary phase survival in which glutamate is mainly required for ROS defense mechanisms (discussed below). The role of *GDH1* gene in glutamate biosynthesis was also investigated in aerobic metabolism [16]. It was observed that yeast cells lacking *GDH1* were unable to divide in acetate/raffinose media, containing ammonia as primary nitrogen source. Furthermore, ^13^C-enrichement experiments confirmed that incorporation of ^13^C into glutamate was nearly undetectable when *gdh1*Δ cells were incubated in [1, 2-^13^C]-acetate/raffinose. The NADP-GDH activity was measured to be less than 15% in *gdh1*Δ cells compared to wild type, confirming the important and primary contribution of Gdh1p in glutamate synthesis under aerobic conditions as well [16].

### *GDH3* transcriptional regulation and the role of the GDH path in ROS-mediated apoptosis

The transcription of *GDH3* occurs extensively during the stationary phase [15, 23]. The activity of Gdh3p presents a 20 to 140-fold increment when cells are grown under aerobic conditions [12]. Under these conditions the majority of the total NADP-GDH activity is attributed to Gdh3p monomers that can contribute up to 70% to the pool, especially when cells enter or remain in aerobic metabolism for several days [15]. Under acetate/raffinose conditions with ammonia as the only nitrogen source, yeast cells lacking *GDH3* gene had a significant impairment in glutamate synthesis [16]. The increase of the NADP-dependent GDH activity observed in *gdh1*Δ mutants was presumably due to Gdh3p that seems to play a prominent role in glutamate metabolism under aerobic conditions [12, 16]. However, glutamate synthesis under aerobic conditions was insufficient and required additionally the activity of Gdh1p [16]. The expression of both *GDH3* and *GDH1* is required to achieve wild-type growth in respiration [12, 16]. The transcriptional regulation of *GDH3* is controlled by carbon sources and not by nitrogen catabolite repression as in the case of *GDH1* [22, 34]. The glucose-repressed expression of *GDH3* is attributed to the condensed chromatin organization of its promoter. Remodeling of the chromatin at the promoter region under non-fermentative carbon sources or under carbon limiting conditions is performed by the SWI/SNF and SAGA complexes [22]. In addition, microarray-based deacetylation maps in yeast revealed that *GDH3* gene is located in a HAST domain (Hda1-Affected SubTelomeric region) where several metabolic genes are glucose-repressed through the action of Hda1 (histone deacetylase 1) [35]. Under diauxic shift, *HDA1* gene is downregulated and through the counteracting activity of Gcn5p acetyltransferase (the catalytic subunit of the SAGA), SWI/SNF complex allows the expression of *GDH3* under respiratory conditions [35]. Gdh3p is stable without being susceptible to post-translational modifications or to phase-specific degradation as Gdh1p. However, it has a lower catalytic capacity in vivo compared to Gdh1p and thus it cannot fulfill the extended needs or substitute the role of Gdh1p during the exponential growth phase in glucose. As such, impairment or lack of *GDH3* does not affect either glutamate production or the survival and growth rates of glucose-grown cells [6, 15]. The low activity of *GDH3* is not attributed to its promoter but to dissimilarities in the amino acid sequence between the two expressed Gdh isoforms. These dissimilarities allows Gdh3p to be more fit for processes that take place in specific cellular compartments like the mitochondria [23]. This is plausible in accelerated evolution which in many instances permits only one of two paralogues to diverge from ancestral functions and to acquire either new or complementary capabilities that favor new metabolic adaptations [13]. Based on this it was observed that the GDH pathway through *GDH3,* and not *GDH1,* is necessary for the resistance to stress-induced apoptosis in stationary-phase yeast cells [23]. Specifically, yeast cells lacking *GDH3* exhibited sensitivity to thermal and oxidative stress as well as oxidative stress-dependent accumulation of ROS that led to apoptotic cell death [23].

Yeast is equipped with mitochondrial enzymes that scavenge free radicals. Among the ROS-scavenging mechanisms, glutathione system (GSH system) is probably the most important intracellular oxidative defense mechanism [23]. The GSH system consists of glutathione (GSH), glutathione reductase and glutathione peroxidase (GPx). Hydrogen peroxide (H_2_O_2_) detoxification is performed by GPx which requires glutathione as reducing power [36]. GSH biosynthesis requires glutamate as primary substrate [36, 37]. Glutamate concentration in stationary yeast cells lacking *GDH3* was 20% less compared to wild type cells [23]. This caused glutamate deficiency subjecting the cells to GSH depletion and thus the observed ROS accumulation that led to apoptosis [23]. A similar phenomenon was observed when stationary *GDH3* mutant yeast cells, were exposed to thermal stress [23]. In addition, it was observed that *GDH3* deletion potentiated ROS generation when yeast cells were treated with ebselen, an antioxidant compound responsible for ROS-mediated cytotoxicity in excessive amounts [38]. Gdh3p was considered the molecular target of ebselen which ceased NADP-GDH activity through the formation of selenyl-sulfide bonds with cysteine residues. It was also observed that the NADP-GDH inactivation might had a critical function in the proteolysis of H3 histone in yeast [38–40], as discussed below.

## The role of GDH path in ammonia production

**Fig. 2:**
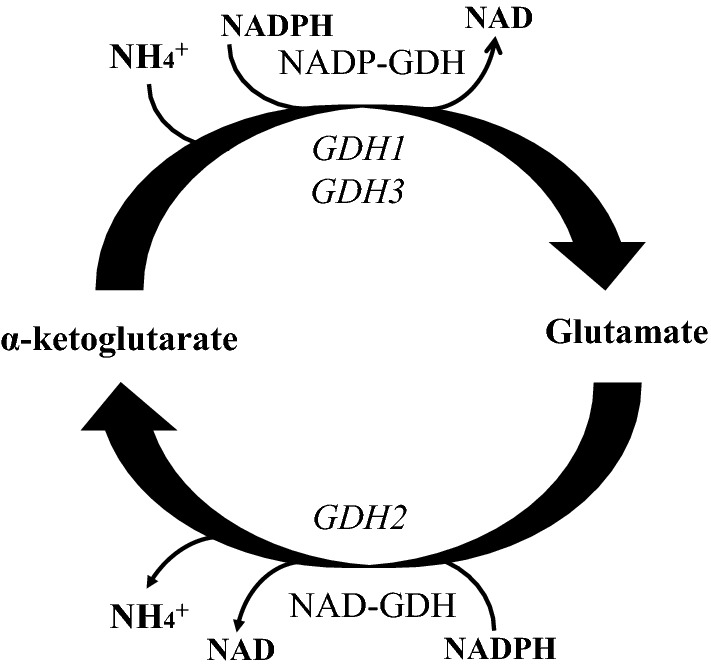
Schematic presentation of the GDH pathway. Synthesis of glutamate occurs through the action of NADP-GDH (encoded by *GDH1* and *GDH3* genes). NAD-GDH activity (encoded by *GDH2*) is responsible for glutamate degradation and release of ammonium and α-ketoglutarate. *GDH2* glutamate dehydrogenase 2

The GDH pathway in *S. cerevisiae* is also responsible for the degradation of glutamate. Contrary to mammals and other organisms, the GDH pathway in yeast presents decoupled functions in terms of glutamate biosynthesis and glutamate catabolism. The NADP-GDH activity of Gdh1 and Gdh3 isoforms is unidirectional and specific for glutamate synthesis, while glutamate catabolism is performed via the action of the oxidizing form of GDH (NAD-GDH) that is also present in the GDH pathway. The NAD-GDH activity in yeast is encoded by *GDH2* gene and catalyzes the oxidative deamination of glutamate to α-ketoglutarate and ammonium [4] (Fig. 2) (2):

2 $${\text{Glutamate }} + {\text{ NAD}}^{ + } \to \alpha {\text{-ketoglutarate}} + {\text{ NH4}}^{ + } + {\text{NADH}}$$

The presence of the NAD-GDH enzyme was initially described with an unclear role in glutamate metabolism [2]. Years later, it was shown that *GDH2* is encoded on chromosome IV of *S. cerevisiae* with a protein-coding sequence containing more than 1000 amino acids, and a strict mitochondrial function that is post-translationally regulated by phosphorylation [41, 42]. As shown, Gdh2p can be converted from an active NAD-dependent glutamate dehydrogenase to an inactive form by phosphorylation through cAMP-dependent and cAMP-independent protein kinases [42]. However, the functional phosphorylation sites have not yet been identified [43].

Initially, it was speculated that yeast cells lacking *GDH1* could use *GDH2* to promote glutamate biosynthesis using ammonia as sole nitrogen source [4]. Through experiments performed both in *S. cerevisiae* and in *A. nidulans* this speculation was gradually abandoned [4]. The NAD-GDH levels were much higher in cells grown in the presence of glutamate compared to those obtained from cells grown in ammonia. Furthermore, the concentration of the NAD co-factor in the cytoplasm was higher under glucose growth conditions, and thus, it was forcing the reaction towards glutamate degradation in order for the NAD–NADH equilibrium to be obtained [4].

Through the enzymatic activity of Gdh2p the breakdown of glutamate provides adequate levels of ammonia in yeast cells [4, 9, 44]. Indeed, the catabolism of glutamate via the NAD-GDH activity is the major pathway of ammonia generation in vivo. In experiments performed under glucose minimal conditions with glutamate as the only nitrogen source, *GDH2* deletion caused limited growth and impaired utilization of the specific amino acid leading to low amounts of intracellular ammonia [4].

The role of *GDH2* in glutamate homeostasis was further examined in cells grown under aerobic conditions [16]. Under acetate/raffinose conditions with ammonia as nitrogen source, the NAD dependent activity of GDH was 20-fold higher compared to that in cells grown in glucose. The disruption of *GDH2* was not deleterious to glutamate homeostasis as expected [16]. In fact, *gdh2*Δ cells presented wild type growth and did not display any deficiencies due to glutamate homeostasis impairment neither under glucose nor under non-fermentable carbon sources [16, 44].

### *GDH2* transcriptional regulation and interaction with *GDH3*

*GDH2* is subjected to transcriptional regulation by glucose [44]. Specifically, it is repressed in glucose and elevated under non-fermentable carbon sources and amino acids as nitrogen source [16, 44]. This strict control is expected due to the anapleurotic role of Gdh2p in Krebs cycle. It is known that Gdh2p replenishes the α-ketoglutarate levels under aerobic conditions [44]. It was considered that the transcriptional activation of *GDH2* would require gene regulators necessary in respiratory growth. However, *GDH2* transcription is independent of the HAP complex, but is regulated by the GATA-type transcriptional activators Gln3 and Ure2 [44, 45]. It was shown that *GDH2* regulation requires two activation and four repression sites present on its promoter. One activation site promotes transcription under glucose starvation [44], while the other one (UAS_NTR_) is implicated in nitrogen catabolite repression [46]. Under nitrogen limitation, Gln3 binds to the UAS_NTR_ and to an adaptor protein (Hfi1) responsible for the integrity of the SAGA transcription activator complex [47]. In the presence of preferred nitrogen sources, the expression of *GDH2* is repressed by the transcriptional regulator Ure2 which sequesters Gln3 into the cytoplasm [4, 45]. It is also found that the expression of *GDH2* is regulated by the concurrent action of Gcn4 and Gln3 [48]. These two regulatory networks have been thought to interact [49–51], putting forward the existence of a physiological relation between Gln3 and Gcn4 [52–54]. Under nitrogen derepressive conditions and amino acid deprivation, Gcn4 and Gln3 form part of a transcriptional complex that binds on *GDH2* promoter and dictates its expression [48].

*GDH2* genetically interacts with *GDH3* and controls stress-induced apoptosis [23]. The role of *GDH2* was investigated in stress-induced apoptosis in stationary phase cells that lacked *GDH3* [23]. It was observed that deletion of *GDH2* gene in a *gdh3*Δ background increased the resistance under thermal or oxidative stress by decreasing ROS accumulation. The apoptosis was suppressed by *GDH2* deletion through the elevated levels of glutamate and glutathione present in the double mutant. Under the tested conditions, deletion of *GDH2* compensated the depletion of intracellular glutamate and glutathione (GSH) followed by stress-induced apoptotic cell death and reinforced further the idea that Gdh2p is responsible only for glutamate catabolism and not its production [23].

## Additional roles of the GDH pathway

### Gdh1p and Gdh3p in nitrogen catabolite repression (NCR)

*Saccharomyces cerevisiae* can utilize a wide variety of nitrogen-based compounds [8]. Despite the broad nitrogen assimilation, not all nitrogen sources can support yeast growth equally well or trigger the same cellular responses [34]. Using these two assumptions, the nitrogen sources in yeast are empirically classified into “rich” or “preferred” and “poor” or “non-preferred” [55]. The “preferred” nitrogen sources are incorporated into glutamate through the GDH or GS–GOGAT pathways and the resulting carbon substrates are readily integrated in metabolism. The “poor” nitrogen sources, including branched-chain amino acids, aromatic amino acids, and methionine, are transferred to α-ketoglutarate by transaminases, forming glutamate [55, 56]. However, the resulting deaminated carbon compounds are converted through the Ehrlich pathway, to non-catabolizable and growth-inhibitory fuse oils [57]. In addition, under “poor” nitrogen sources, activation of the general control of amino acid biosynthesis (GAAC) mechanism is observed. GAAC activation is mediated by the transcription factor (TF) Gcn4 responsible for the expression of a large number of genes involved in amino acid biosynthesis [55, 58]. Although we recognize the importance of GAAC mechanism, we consider that a more detailed discussion on the regulation of GAAC under “poor nitrogen” sources has been reviewed elsewhere [55, 56] and exceeds the purposes of this review.

The preferential expression of genes involved in nitrogen metabolism is primarily controlled in *S. cerevisiae* by a transcriptional mechanism known as nitrogen catabolite repression (NCR). Through NCR, baker’s yeast can downregulate the expression of genes involved in the utilization of non-preferable nitrogen sources when preferable nitrogen compounds are available [11, 59]. NCR mechanism comprises four transcription factors (Gln3, Gat1, Dal80 and Gzf3) with a zinc-finger DNA binding domain that recognizes the GATA motif in the promoter of target genes [60]. NCR in yeast is additionally controlled by Ure2, a transcription factor responsible for the translocation of the four latter TFs into the nucleus [61]. According to different experimental studies, in the presence of ammonium, Ure2 binds Gln3 in the cytoplasm and prevents its translocation to the nucleus [60, 62]. This allows Dal80 and Gzf3 to repress the expression of NCR-sensitive genes involved in utilization of alternative nitrogen sources [60]. Ure2 releases Gln3 under nitrogen starvation or in the presence of non-preferable nitrogen sources [34], and upon release, Gln3 translocates into the nucleus. Gln3 and Gat1 act together, mediating the transcription of NCR-sensitive genes [63, 64].

NCR has been described to respond to glutamine and glutamate deficiencies through the activation of Gln3 or Gat1 respectively, while ammonium is considered a distinct signal for NCR that acts independently of the glutamine and glutamate levels [34]. In addition, NCR is controlled by TOR signaling that mediates cell growth and metabolism under different nitrogen and carbon sources. Experimental studies showed that inhibition of TORC1 resulted in nuclear accumulation of Gln3 and Gat1 and transcriptional depression of NCR-sensitive genes [52, 54]. However, as discussed below, growing literature suggests that TORC1 is not the only regulator of these specific GATA-type transcription factors [34, 61, 65, 66]. Specifically, *GDH1* was found to have a prominent role in nitrogen-responsive activities [34]. Under nitrogen repressive conditions the derepression of NCR-sensitive genes, like *GAP1* (general amino acid permease 1), was due to the drastic effect that *GDH1* deletion had on the localization and function of the essential GATA-type activator, Gat1. It was observed that lack of *GDH1* mediated the accumulation of Gat1 into the nucleus and thus derepressed the transcription of *GAP1* gene. This finding also questioned the role of *GDH1* on the other GATA-type activator, Gln3. Upon *GDH1* deletion, a highly derepressed expression of *DAL5*, a NCR-sensitive gene that requires both Gat1 and Gln3 for its expression, was observed. *GDH1* deletion caused ammonium accumulation as expected, but surprisingly did not affect the subcellular distribution and the concentrations of glutamine as well as glutamate [34]. This suggested that the Gat1 and Gln3-mediated expression of *DAL5* and *GAP1* was independent of the glutamine/glutamate levels. Indeed, the *GDH1*-based repression of *DAL5* and *GAP1* was strongly correlated with the activity of Gdh1 enzyme per se [34]. As shown, *GAP1* gene was depressed in yeast strains encoding a mutation responsible for the inactivation of the catalytic site of Gdh1 (Gdh1p^K110L^). On the contrary, the transcriptional repression of *GAP1* was strong when the NADP-GDH activity was restored by expressing the *GDH3 gene,* and partially restored, when the bacterial *GDHA* was expressed in *gdh1* mutant strains [34]. However, the signals for the GDH-dependent negative regulation of NCR-sensitive genes require further investigation.

### Gdh1 and Gdh3 enzymes in chromatin regulation in yeast

The role of Gdh1p in transcriptional silencing was found to be crucial through the proteolysis of H3 histone in yeast (“H3-clipping” in the N-tail) [40]. This effect has been described previously in animal tissues [39, 67]. In yeast however, the proteolysis of H3 histone had been observed initially in sporulating and stationary phase cells, through the action of the vacuolar serine protease Prb1 [68]. Recent studies revealed that deletion of *GDH1* gene increased “H3-clipping” in log phase cells revealing an inhibitory role of Gdh1p on the N-terminus cleavage of H3 [40]. Another observation was that Gdh1p mediated the silencing of sub-telomeric regions through the recruitment of SIR complex [40]. This highlighted the association of an enzyme primarily described in metabolism, with epigenetic processes. Specifically, *GDH1* deletion led to decreased binding of Sir2 protein on the telomeres, causing elevated transcript levels of genes affected by the loss of the SIR complex [40]. *GDH1* was found to regulate chromatin through its catalytic activity [40]. Specifically, upon *GDH1* deletion, the elevated levels of α-ketoglutarate, and not those of NADH, resulted in the observed telomeric silencing defects [40]. The authors described  physical association of Gdh1p on specific telomeric loci that were under the transcriptional control of SIR complex. Additional experiments showed that α-ketoglutarate levels changed when Gdh1p was depleted from the nucleus. This suggested that Gdh1p possibly affects the levels of α-ketoglutarate at the SIR-regulated telomeric loci.

The intracellular levels of α-ketoglutarate seems to have a “mechanistic” role also in other organisms. Experiments in mice and nematodes (*Caenorhabditis elegans*) showed that modulation of the intracellular levels of α-ketoglutarate interfere with gene transcription and longevity [52, 53, 69]. Moreover, modulation of α-ketoglutarate levels found to interfere with the epigenetic state and cellular fate of mouse embryonic stem cells [70]. *GDH3* is also implicated in both metabolism and chromatin configurations [40]. Specifically, loss of telomeric silencing was observed in the double *gdh1*Δ *gdh3*Δ mutant grown both in ethanol and glucose. Under ethanol conditions deletion of either *GDH* gene led to mild silencing defects. Also, increased expression of *GDH3* partially complemented the *gdh1*Δ phenotype. This demonstrated overlapping functions of the two isoforms that affect heterochromatin regulation [40].

### The contradictory roles of *GDH1* and *GDH2* in cold-growth defects in yeast strains

The role of *GDH1* and *GDH2* found to be contradictory when investigated in yeast strains under cold-growth conditions [71]. Using recombinant strains of *S. cerevisiae*, it was observed that overexpression of *GDH1* had detrimental effects on yeast growth at 15 °C creating a cold-sensitive yeast phenotype. On the contrary, overexpression of *GDH2* was favoring yeast growth providing a growth advantage in the same conditions [71].

The GDH pathway interferes with the recycling of the essential coenzymes NADH–NAD by controlling the levels of α-ketoglutarate [72]. NADH–NAD homeostasis is crucial for proper cellular responses under environmental changes [73]. Ballester-Tomás et al. [71], suggested that growth temperatures below the optimal require a proper redox NADH–NAD balance that was probably disrupted through the overexpression of *GDH1*. However, under the examined conditions, the overexpression of *GDH2* seemed to regulate this NADH–NAD imbalance, through the increased oxidation of NADH [71]. This was in accordance with previous studies reporting that increased NADH oxidation altered the distribution of metabolic fluxes and sustained yeast growth at suboptimal temperature conditions [74, 75]. Furthermore, an additional study showed the activity of NAD-related genes governs cold growth in yeast and that *GDH2* is a cold-growth favoring gene [76].

Shifts to low growth temperatures create increased intracellular H_2_O_2_ levels and induced expression of antioxidant genes implicated in glutathione synthesis [77, 78]. Under cold-growth conditions, Ballester-Tomás et al. [71] showed that concurrent ectopic overexpression of *GDH1* and *GDH2* compensated the observed accumulation of ROS. The authors suggested that this is consistent with the role of *GDH1* and *GDH2* in glutamate synthesis and its possible implication to oxidation stress defense through the glutathione system. Specifically, glutamate can prevent cold-induced ROS accumulation through the synthesis of glutathione that requires glutamate as a precursor molecule and serves in ROS removal.

### Implications of the GDH pathway in actin cytoskeleton, endocytosis and iron deficiency

Proper function of Gdh3p has a role in the sphingolipid-dependent suppression of reduced viability on starvation (*RVS*) defects that include inability to grow under nutrient starvation or osmotic stress [79]. *RVS* genes encode the calmodulin-binding, actin-associated, amphiphysin-like lipid raft proteins Rvs167 and Rvs161 which are not essential for yeast viability. However, the Rvs proteins contain a conserved BAR domain that appears to regulate endocytosis, actin cytoskeleton structure and nuclear events. As such, under nutrient depletion, recessive mutations or total loss of the *RVS* genes, cause growth abnormalities and cytoskeletal and endocytosis defects [80, 81]. Specifically, it was observed that the proper function of Gdh3p altered the growth defects provoked upon *RVS* and *SUR4* deletions in yeast [79]. *SUR4* in yeast encodes an elongase involved in fatty acid and sphingolipid biosynthesis. As it seems, Gdh3p participates in a sphingolipid-dependent manner to the restoration of growth under glucose starvation. In addition, Gdh3p was found to physically interact with three domains of the Rvs167p which forms with Rvs161p a complex, that regulates cell polarization, actin cytoskeleton, endocytosis and cell cycle [79]. The role of Gdh3p in that configuration is not known and it will be interesting to examine whether its catalytic activity is primarily involved.

Iron deficiency has a significant impact on amino acid biosynthesis in yeast [10]. It has been observed that many transcripts involved in amino acid metabolism are regulated during iron deficiency and that most of the amino acids affected include an iron-dependent step in their synthesis [82]. Transcriptomic analysis showed that upon iron deficiency, genes implicated in the GS–GOGAT pathway were downregulated while genes of the GDH pathway were upregulated [10]. Specifically, yeast cells exhibited a sixfold downregulation of *GLT1* (encodes the NAD-dependent glutamate synthase–GOGAT) as opposed to *GDH3* that was 4.5-fold upregulated under the same conditions [10]. This made GDH pathway an iron-independent pathway compared to GS-GOGAT that is iron-dependent. The reason for this differentiation lies on glutamate synthase that is a Fe–S requiring enzyme. Indeed, under iron deficiency the activity of glutamate synthase presents a 20-fold decrease [82]. As already mentioned, the vast majority of nitrogen-containing molecules in yeast acquire their nitrogen from glutamate and to a lesser extent from glutamine, so continuous synthesis of these two amino acids is critical and highly regulated under iron-limiting conditions.

### Can the GDH pathway provide additional information on the role of GABA shunt in *Saccharomyces cerevisiae*?

Although the purpose of this review focuses on the known pleiotropic effects of the GDH pathway, we consider that it will be beneficial for the reader to briefly describe the GABA shunt in *S. cerevisiae* and suggest possible interactions with the GDH pathway. The GABA shunt is a pathway utilized in many organisms for the conversion of glutamate to succinate, via the formation of γ-aminobutyric acid (GABA) and succinic semialdehyde [83, 84]. The pathway involves the decarboxylation of glutamate by Gad1p and the sequential action of GABA aminotransferase (Uga1p) and succinic semialdehyde dehydrogenase (Uga2p) for the production of succinate. In *S. cerevisiae*, the expression of *UGA1* and *UGA2* is upregulated by GABA, whereas the expression of *GAD1* has been linked with calcium levels since Gad1p is a calmodulin-binding protein [85, 86]. In addition, the expression of *GAD1* is proposed to be regulated by TOR signaling [87]. Experimental studies showed that yeast utilizes and stores GABA as nitrogen source and can transport it using the inducible GABA-specific transport protein (encoded by *UGA4*), general amino acid permease Gap1 and proline-specific permease Put4 [88–90].

GABA shunt is still poorly understood in yeast, and has received less attention compared to other studied nitrogen pathways in *S. cerevisiae* [84, 87]. However, it is suggested that it has a role in cellular oxidative stress defense and is upregulated during the stationary phase, under nitrogen starvation [91]. Studies performed in yeast cells have shown that expression of *GAD1* provided increased tolerance to oxidative agents compared to *GAD1* mutant strains, while deletion of *UGA1* and *UGA2* rendered the cells hypersensitive compared to wild type strains [86]. The authors observed that the role of GABA shunt in oxidative stress was strictly dependent on the presence of the intact GABA catabolic pathway, and suggested the importance of GABA as a signal to stress responses, as described in other organisms [92]. A recent study attributed the strong growth inhibition of *UGA2* yeast mutants, to the accumulation of succinate semialdehyde that is considered a potential toxic intermediate of GABA catabolism [93]. Finally, mutations in the GABA shunt genes resulted in yeast strains with reduced heat-stress tolerance compared to wild type [84]. The heat susceptibility observed in the mutant strains was correlated with the high intracellular ROS concentrations produced under the same growth conditions.

**Fig. 3:**
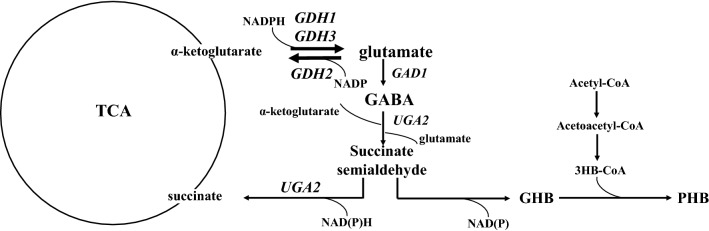
GABA catabolism and production of polyhydroxybutyrates (PHBs) in *S. cerevisiae*. The figure was adapted from Fig. 1 [87]. PHBs production starts with the reduction of succinate semialdehyde by γ-hydroxybutyric acid dehydrogenase to produce γ-hydroxybutyric acid. γ-hydroxybutyric acid and 3-hydroxybutyrate are polymerized by poly(3-hydroxybutyrate-co-4-hydroxybutyrate) synthase. 3HB formation from acetyl-CoA involves acetoacetyl-CoA thiolase and 3HB-CoA dehydrogenase. *GDH1* glutamate dehydrogenase 1, *GDH2* glutamate dehydrogenase 2, *GDH3* glutamate dehydrogenase 3, *GAD1* glutamate decarboxylase, *UGA2* succinate semialdehyde dehydrogenase, *GHB* γ-hydroxybutyric acid, *3HB* 3-hydroxybutyrate, *P(3HB-co-4HB)* poly(3-hydroxybutyrate-co-4-hydroxybutyrate)

As discussed above, the GDH pathway has a role in cellular ROS defense in stationary phase cells and is linked with NADPH availability and NADH oxidation during yeast growth under suboptimal conditions. Although to our knowledge, there is no direct evidence of crosstalk between the GDH pathway and the GABA shunt, we speculate that the GDH pathway may interfere with the decarboxylating step of the GABA shunt by controlling the available glutamate via the action of Gdh1, Gdh2 and Gdh3 proteins under specific conditions. We also speculate that the GDH pathway may also control the fate of the last reaction of the GABA shunt, which involves the degradation of GABA to γ-aminobutaric acid, instead of succinate, as previously described in *S. cerevisiae* (Fig. 3). γ-aminobutaric acid is a substrate produced from GABA degradation through the action of γ-aminobutaric acid dehydrogenase. γ-aminobutaric acid can be utilized for the production of polyhydroxybutyrates (PHB), a type of complex macromolecules that accumulate as discrete granules in the cytosol of many microorganisms and serve as energy-storage molecules under imbalanced nutrient conditions [94]. During the last decades the properties of PHBs have been extensively investigated in bacteria [95–97]. Besides their role as carbon storage molecules, they were described as biopolymers with similar properties to common plastics with the unique ability to biodegrade [94]. Efforts to utilize bacteria for the production of PHBs in an industrial scale resulted in low yields of PHBs, leading to the search of other biotechnological platforms [87]. As discussed above, GDH pathway in yeast has an anapleurotic role and replenishes α-ketoglutarate for the production of succinate in Krebs cycle. GABA shunt also facilitates Krebs cycle by providing succinate via GABA catabolism. If the fate of GABA catabolism, favoring the production of γ-aminobutaric acid over succinate, is partially controlled by GDH pathway, then formation of PHBs via GABA under specific conditions, opens new opportunities to use *S. cerevisiae* for PHB-related compounds.

## Conclusions

The GDH pathway has a key role in glutamate homeostasis and ammonium assimilation in yeast cells. Synthesis of adequate levels of glutamate utilizing two GDH enzymes with different metabolic activities reflects a unique evolutionary advantage of *S. cerevisiae.* This ability can fulfill the requirements of both fermentative and respiratory metabolic growth under ammonium excess. This leads to an efficient exploitation of the carbon sources available at each given growth phase. The other essential adaptation in the GDH pathway of *S. cerevisiae* is the expression of a third GDH enzyme localized in the mitochondria that is primarily responsible for the degradation of glutamate and the anapleurosis of Krebs cycle by providing α-ketoglutarate. The presence of three GDH enzymes in yeast assures rapid nitrogen assimilation and glutamate biosynthesis during fermentation and promotes the wise use of α-ketoglutarate without disturbing the proper function of Krebs cycle under respiratory conditions.

**Fig. 4:**
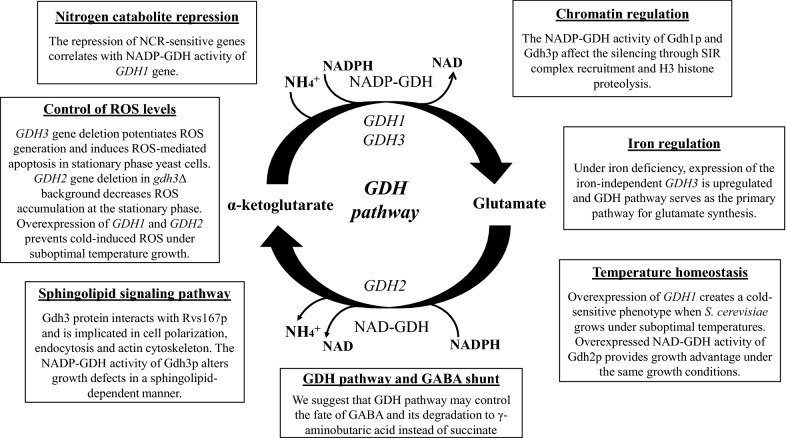
The pleiotropic functions of GDH pathway in *S. cerevisiae.* The enzymatic activity of Gdh1p, Gdh3p and Gdh2p regulates glutamate homeostasis and influences other essential cellular processes

During the past years, cumulative evidence showed that the GDH pathway affects a broader range of cellular activities (Fig. 4). This is an established example showing that cellular metabolic status coordinates the correct function of different cellular compartments. Indeed, the GDH pathway through the action of Gdh1p and Gdh3p showed a strong positive effect on epigenetic processes that promote telomeric silencing. Additionally, the metabolic capacity of Gdh1p and Gdh3p isoforms is essential in the regulation of α-ketoglutarate levels that seems to be the signal also for the NCR-regulated gene expression. These findings demonstrate new roles for the two conserved NADP-GDH enzymes that were primarily described for their role in glutamate production. Additionally, the increased metabolic activity of NADP-GDH through *GDH1* overexpression seemed to alter the NAD–NADH levels and thus yeast growth under suboptimal temperatures.

The GDH pathway was also implicated in the regulation of ROS under different conditions. The lack of *GDH3* provoked yeast cells sensitivity to thermal and oxidative stress leading to apoptotic cell death due to ROS accumulation. Furthermore, treatment of *gdh3*Δ yeast strains with the drug ebselen, potentiated ROS generation and possibly affected the function of NADH-GDH as a histone H3 protease, as it was also observed in chicken [67]. *GDH2* was found to genetically interact with *GDH3* and *GDH1* and regulate ROS levels. Finally, the GDH pathway was found to be the iron-independent pathway that regulates glutamate production in yeast cells grown in iron limiting conditions. The importance of the GDH pathway in glutamate homeostasis, nitrogen assimilation and its role to various cellular functions is not restricted only to yeast cells. It has been observed that glutamate production through the appropriate function of GDH pathway has a role in the mitochondrial retrograde signaling that affects changes in nuclear gene expression related to nutrient sensing and TOR signaling [26, 98], aging [99, 100] metabolism [20] and as recently shown, in different types of cancer [101–108]. Furthermore, malfunction of the GDH pathway has been implicated in several other human diseases including diabetes [109–111], neurodegenerative disorders [112], as well as congenital syndromes that affect mainly children [113].

*Saccharomyces cerevisiae* is a model organism frequently used for biotechnological purposes [114–117]. Being a successful biotechnological tool for many decades, yeast still remains a cell factory that can be used for further challenges regarding genetic engineering and maintenance of redox balances, biosynthesis of basic metabolites and control of biosynthetic pathways [114]. Hosting three different and independent GDH enzymes makes *S. cerevisiae* an excellent system for better understanding the GDH-derived glutamate and its fate on post translational modifications, epigenetics, chromatin regulation, signaling, oxidative stress defense mechanisms and efflux processes essential for redox homeostasis. Finally, the decoupled functions of the GDH pathway in *S. cerevisiae*, in terms of glutamate biosynthesis and catabolism can be used as a model to address important biotechnologically related questions on the role and regulation of organic substrates like α-ketoglutarate. This seems to be important due to the significance of α-ketoglutarate in stress responses, lifespan extension, cellular senescence, tumor suppressing conditions and human diseases.
